# PAX8 is a novel marker for differentiating between various types of tumor, particularly ovarian epithelial carcinomas

**DOI:** 10.3892/ol.2013.1121

**Published:** 2013-01-10

**Authors:** LI XIANG, BEIHUA KONG

**Affiliations:** Department of Obstetrics and Gynecology, Qilu Hospital, Shandong University, Jinan 250012, P.R. China

**Keywords:** PAX8, tumor marker, ovarian epithelial carcinoma

## Abstract

Paired-box gene 8 (PAX8) encodes a transcription factor associated with important roles in embryogenesis and disease, and is a member of the PAX gene family. PAX8 has been demonstrated to be crucial in determining cell fate during the development of the thyroid, kidney, brain, eyes and Müllerian system and regulates expression of the Wilms’ tumor suppressor gene (WT1). Several previous studies have reported that PAX8 is expressed at high levels in specific types of tumor, including thyroid and renal carcinomas and pancreatic neuroendocrine tumors. In addition, PAX8 has been reported to be useful for the detection and differential diagnosis of ovarian carcinoma. The consistency of PAX8 staining in epithelial ovarian carcinomas (EOCs) and the fallopian tube has provided morphological evidence that EOC may originate from the fallopian tube. The molecular mechanism of PAX8 in the carcinogenesis of these tumors remains unclear and requires further studies.

## Contents

1. Introduction
2. Expression of PAX8 in various types of tumor
3. PAX8 detection in ovarian epithelial cancer
4. Conclusions

## 1. Introduction

Paired-box genes (PAX) encode a family of nine well-characterized paired-box transcription factors (PAX1-9), which are important for embryogenesis and disease (1). PAX proteins have been implicated as regulators of organogenesis and key factors in maintaining pluripotency of stem cell populations during development. Among these PAX genes, PAX8 has been hypothesized to be crucial in determining cell fate during the development of the thyroid, kidney, brain, eyes and Müllerian system and regulates expression of the Wilms’ tumor suppressor gene (WT1) (2–7). A number of previous studies have identified that PAX8 is not only expressed in the aforementioned organs but also found at high levels in specific types of tumor, including thyroid and renal carcinomas and pancreatic neuroendocrine tumors (8–11). In addition, PAX8 has been found to be expressed at high levels in ovarian carcinomas and serous effusions (3,12), indicating that PAX8 detection may prove useful in the clinic (Table I).

## 2. Expression of PAX8 in various types of tumor

A number of studies have found that PAX8 is important for the development of various types of tumor (8–10). Together with thyroid transcription factor (TTF)-1 and TTF-2 (FoxE1), PAX8 is markedly associated with thyroid gland organogenesis. PAX8 expression has also been identified in thyroid carcinomas (10,13,14). Tacha *et al* found that PAX8 was expressed in 90% of thyroid cancer cases (15). In addition, follicular thyroid carcinoma (FTC) accounts for 10–20% of all thyroid cancers and up to 40% of cause-specific mortalities. Notably, the resulting fusion protein, PAX8/peroxisome proliferator-activated receptor (PPAR)-γ, has been found in ∼50% of FTC cases (13,16,17). Chromosomal translocations involving PAX5 and PAX8 genes in thyroid cancer indicate that PAX genes have an oncogenic capacity when constitutively expressed, either as part of a fusion gene or as a whole gene (10,18).

Previously, PAX8 was found to be markedly associated with renal tumors (19,20). Renal cell carcinomas (RCC) stained positive for PAX8 in 90% of the cases studied and 100% of normal kidney samples stained PAX8-positive (15). Knoepp *et al* reported that immunoreactivity for PAX8 and PAX2 was observed in 88 and 83% of 24 cytology specimens, respectively. The presence of either PAX8 or PAX2 immunostaining revealed a total sensitivity of 92%, indicating that PAX8 and PAX2 are useful adjuncts for confirmation of RCC diagnoses in cytology specimens (9). Collecting duct carcinoma (CDC) is a relatively rare but aggressive form of renal malignancy with variable morphological features and lacks a suitable marker for detection. A previous study found that all CDC cases were positive for PAX8. The immunoprofile of PAX8^+^/p63^−^ is consistent with the diagnosis of CDC with a sensitivity of 85.7% and specificity of 100%. By contrast, a PAX8^−^/p63^+^ profile indicates upper urinary tract transitional cell carcinoma (UUC) with a sensitivity of 88.2% and specificity of 100%. The nephric lineage restriction of PAX8 indicates a renal tubular rather than an urothelial differentiation in CDC according to the inverse PAX8/p63 expression observed in CDC and UUC (20).

Furthermore, PAX8 has been recognized as a potential immunohistochemical marker of pancreatic neuroendocrine tumors (8,21). It was reported that among well-differentiated neuroendocrine tumors, only tumors from the pancreas were PAX8-positive (14/25, 56%) whereas no cases of pulmonary, ileal, duodenal, rectal or ovarian well-differentiated neuroendocrine tumors were positive for PAX8 (8). The observation that pancreatic well-differentiated neuroendocrine tumors frequently express PAX8 may be useful for distinguishing pancreatic primary tumors from tumors of other anatomical sites. By contrast, PAX8 expression is not specific for pancreatic origin in poorly differentiated neuroendocrine carcinomas, whereas it is observed in extrapancreatic poorly differentiated neuroendocrine carcinomas, indicating that PAX8 expression is not markedly associated with poorly differentiated neuroendocrine carcinomas. Sangoi *et al* found that PAX8 was positive in 74% of primary pancreatic neuroendocrine tumors and PAX8 expression did not correlate with World Health Organization categorization, grade, size, functional status or the presence of liver or lymph node metastases (22). Among liver metastases, only pancreatic neuroendocrine tumors (20/31, 65%) were PAX8-positive, whereas no cases of ileal, pulmonary, duodenal and rectal neuroendocrine tumor metastases were PAX8-positive. Overall, PAX8 is expressed in primary and metastatic pancreatic well-differentiated neuroendocrine tumors, enabling reliable differentiation between pancreatic and ileal and pulmonary well-differentiated neuroendocrine tumors using immunostaining methods.

Ovarian cancer is one of the most lethal forms of cancer in females and currently lacks useful markers and efficient screening methods (23), due to the complexity of variable subtypes. At present, the most common marker used in monitoring therapy of this disease is CA-125; however, this marker is not specific and sensitive enough to be useful as a screening test, with serum values in the normal range in half of patients with stage I disease (24,25). Considerable efforts have been made to identify suitable markers for detection and differentiation between various forms of ovarian cancer. In 2003, analysis of the PAX8 gene by DNA microarray revealed high expression in ovarian cancer. Using the prediction analysis of microarrays (PAM) method, the expression of 61 genes was analyzed in 68 breast and 57 ovarian carcinoma samples and PAX8 expression was found at higher levels in ovarian compared with breast cancer (26). Consistent with these observations, microarray analysis performed by Bowen *et al* found that PAX8 was highly expressed in EOCs, whereas it was absent from the ovarian surface epithelia of healthy individuals. In addition, the authors observed that PAX8 was localized to the nucleus of non-ciliated epithelia in simple ovarian epithelial inclusion cysts and in 3 epithelial ovarian cancer subtypes (serous, endometrioid and clear cell). PAX8 was also found to be expressed in the non-ciliated secretory cells of healthy fallopian tube mucosal linings but not in the adjacent ciliated epithelia (27). Laury *et al* reported that PAX8 staining was present in 99% of high-grade serous ovarian carcinomas and 100% of low-grade ovarian carcinomas and serous borderline tumors (28). Tacha *et al* identified that 79% of ovarian cancers expressed PAX8 (15). These observations indicate that PAX8 may prove useful for the detection of ovarian cancers.

## 3. PAX8 detection in ovarian epithelial cancer

### Differential diagnosis of ovarian and breast cancer

The ovary is a common site for formation of metastases and the breast is one of the most common sources. Ovarian and breast cancers develop from hormonally responsive tissues, comprise various histopathological subtypes and exhibit considerable variability in clinical manifestations and prognosis. Metastatic breast carcinoma is known to morphologically mimic primary ovarian carcinoma, resulting in difficulty in distinguishing between these forms of cancer. A previous study using microarray analysis revealed that PAX8 and EPAC are expressed at higher levels in ovarian compared with breast cancer (26). Previously, WT1 was considered to be a suitable marker to distinguish metastatic breast cancer from ovarian carcinoma. However, WT1 was later observed in focal breast cancer, causing false positive results. By contrast, PAX8 was stained in none of the breast and almost all ovarian cancer samples, indicating that PAX8 is a more superior marker for the differential diagnosis of ovarian and breast cancer (11).

### Detection of metastatic ovarian carcinoma

Ovarian cancers are frequently associated with metastases, which are commonly found in peritoneal fluids (29). However, reactive mesothelial cells in effusion specimens are known to morphologically mimic ovarian serous carcinoma, making diagnosis difficult (30). Previously, calretinin was identified as a reliable immunohistochemical marker for mesothelial cells and WT1 was hypothesized to be useful in the diagnosis of ovarian serous carcinoma. However, mesothelial cells have also been found to exhibit immunoreactivity against WT1 (31). Recently, PAX8 was revealed to be expressed at high levels in EOC. By contrast, mesothelial cells stained negative against PAX8 (40). PAX8-positive, calretinin-negative staining appears to be highly specific and sensitive for detecting metastatic ovarian serous carcinoma in cytological preparations and may prove useful for distinguishing these cells from mesothelial cells in fluid cytology (32). Tong *et al* reported that PAX8 was detected in 70 and 68.8% of metastatic carcinomas of the ovary and endometrium in serous effusions, respectively (12). In addition, our previous study found that detection of PAX8 is useful for recognition of metastatic carcinomas in pelvic washings, particularly in cases with suspicious cytology (33).

### EOC originates from the fallopian tube

EOC is one of the most common forms of ovarian cancer and its etiology and origin have been studied for a number of years. A number of hypotheses on the origin of EOC have been presented; however, none of these mechanisms have been officially recognized. A traditional hypothesis on the origin of EOC indicates that EOCs arise from the single layer of cells found surrounding the ovary, referred to as ovarian surface epithelia (OSE), which are the modified coelomic or peritoneal mesothelia that form a single layer of flat-to-cuboidal cells covering the ovary (15,34,35). By contrast, additional studies have reported that EOC originates from the fallopian tube (36–39). Bowen *et al* observed that PAX8 was localized to the nucleus of non-ciliated epithelia in simple ovarian epithelial inclusion cysts and in three epithelial ovarian cancer subtypes (serous, endometrioid and clear cell). The authors found that PAX8 was also expressed in the non-ciliated, secretory cells of healthy fallopian tube mucosal linings but not in the adjacent ciliated epithelia (27), consistent with our own study (40). The findings suggested a possible correlation among EOCs, OSE and the fallopian tube. Marquez *et al* previously reported that when compared with normal ovarian epithelial brushings, alterations in microarray gene expression profiles of serous tumors correlated with those in normal fallopian tube (P=0.0042) but not in other normal tissues (41), indicating that EOCs not only imitate the phenotype of other differentiated epithelia of the female reproductive tract, but also their gene expression profiles. Morphological and genetic analyses indicate that expression levels of PAX8 are consistent with the hypothesis that EOC originates from the fallopian tube. At present, these observations remain controversial and challenging and additional studies should be performed to clarify the molecular mechanisms of PAX in the origin and carcinogenesis of EOC.

## 4. Conclusions

PAX8 is not only crucial for determining cell fate during the development of the thyroid, kidney and Müllerian system, but has also been found to be expressed at high levels in thyroid and renal carcinomas and pancreatic neuroendocrine tumors. Recently, PAX8 has been used in the detection and differential diagnosis of ovarian epithelial carcinomas. Consistencies in PAX8 staining between EOC and the fallopian tube indicate that EOC may originate from the fallopian tube. However, the molecular mechanism of PAX8 in the carcinogenesis of these tumors remains unclear and requires further analysis.

## Figures and Tables

**Table I:** Immunohistochemical expression of PAX8 in various forms of carcinoma.

Histotype	% positive	Refs.
Thyroid carcinoma	79–90	10,13,14,15
Renal cell carcinoma	88–100	9,15,19,20
Pancreatic neuroendocrine tumors	56–74	8,21,22
Ovarian carcinoma		
Primary	79–100	3,15,27,28
Metastatic	70–96	12,32,33

PAX8, paired-box gene 8.
